# Photoplethysmography for the Assessment of Haemorheology

**DOI:** 10.1038/s41598-017-01636-0

**Published:** 2017-05-03

**Authors:** Haneen Njoum, Panayiotis A. Kyriacou

**Affiliations:** Research Centre for Biomedical Engineering, City, University of London, EC1V, UK

## Abstract

Haemorheology has been long identified as an early biomarker of a wide range of diseases, especially cardiovascular diseases. This study investigates for the first time the suitability of Photoplethysmography (PPG) as a non-invasive diagnostic method for haemorheological changes. The sensitivity of both PPG components (AC and DC) to changes in haemorheology were rigorously investigated in an *in vitro* experimental setup that mimics the human circulation. A custom-made reflectance PPG sensor, a pressure transducer and an ultrasonic Doppler flowmeter were used to map changes in flow dynamics and optical responses in an arterial model. The study investigated the effect of shear rates by varying fluid pumping frequencies using 4 set-points and the effect of clot formation using a chemical trigger. Both PPG_AC_ amplitudes and PPG_DC_ levels showed significant (p < 0.001) changes during the increase in shear rates and an immediate change after thromboplastin activation. The findings highlight that PPG has the potential to be used as a simple non-invasive method for the detection of blood characteristics, including disaggregation, radial migration and cross-linking fibrin formations. Such capability will enable the assessment of the effects of clotting-activators and anticoagulants (including non-pharmacological methods) and might aid in the early non-invasive assessment of cardiovascular pathologies.

## Introduction

Haemorheology is the study that combines blood flow mechanics and haematology. Evidence from the literature confirms that impaired haemorheological properties are associated with pathologies such as sepsis^1^, sickle cell anaemia^2, 3^, diabetes^4^, gynaecologic malignancy^5^ and cardiovascular diseases^6–9^. The early manifestation of impaired haemorheological properties makes it a potential biomarker for the detection and screening of a number of pathologies.

Despite the emphasis of the literature on the link between haemorheology and disease, routine clinical tests do not tend to include haemorheological assessments. Particularly in cardiovascular patients, there is a plethora of evidence that points to the association of hyperviscosity in the increased risk of complications^6, 8–11^. Platelet activation is also a major factor in the progression of atherosclerosis and lesion formation^12–15^. From a research point of view, various tests and devices have been developed over many decades to assess blood rheology and platelet function *in vitro*. These can be summarised as blood viscosity measurements^16^, blood clotting and platelet adhesion^17^, and deformation or rigidity index^18^. Rosencranz and Bogen highlighted in their article that most of the experience with viscosity instrumentation has been outside the biomedical field and in the manufacturing of paint, solvents, petrochemicals, and polymers^16^. In addition, many of the available viscometers are only used for research and very few for clinical use. Using blood viscosity tests rather than plasma viscosity tests has also been limited, mainly due to the concern of users being exposed to blood, and also due to the variability in the measurements as manual viscometers are operator-dependent. These factors may be some of the reasons why blood viscosity is being overlooked as an integral part of current cardiovascular profiling. Laboratory tests of platelet function, such as bleeding time, light transmission platelet aggregation, Lumi aggregometry, impedance aggregometry and platelet activation conducted by flow cytometry are traditionally utilised for managing platelet and haemostatic disorders, but their use is limited to specialised laboratories. In a similar way, deformation and rigidity of erythrocytes tests are limited for research purposes only. More recently a point-of-care platelet function testing became available^19, 20^, however, the method is performed using a static sample. This method does not take into account the haemodynamic forces in the circulation, plus it requires professional training in order to obtain the data and interpret it and has no potential to be utilised *in vivo* for continuous detection of rheological changes.

This work is primarily focused on investigating the sensitivity of the optical technique Photoplethysmography (PPG) and its components (AC and DC) to changes in hemorheological characteristics. PPG is an optical technique, commonly utilised in the estimation of blood oxygen saturation (SpO_2_) in pulse oximetry^21, 22^. PPG is also one of the main techniques used in wearables such as smart watches used for heart rate measurement and estimations of other physiological parameters^23^. The PPG_AC_ component is known to be synchronised with the heartbeat, while the PPG_DC_ component tends to be associated with neurohormonal factors^24^. Researchers have investigated the PPG beyond pulse oximetry such as the relation of the PPG with venous saturation^25^. Also, investigations of PPG during vascular occlusions^26^, sympathetic blockade^27^ and during exposure to thermal stresses^28, 29^ have also been reported. Following rigorous literature search, this seems to be the first study that investigates PPG in relation to hemorheological properties. This approach aims to provide a better understanding of the factors that contribute to changes in the PPG signal and the role of light scattering and absorption in the AC and DC PPG components. Also, in this study, we aim to prove the concept that PPG has the potential to assess hemorheological properties non-invasively, which if successful, will enable the detection and/or screening of pathologies at an early stage and aid in the pharmacological and non-pharmacological management of the disease. In this study, two major factors, and their effect on the PPG were investigated. Firstly, the effect of shear rates by altering pumping frequencies and secondly the role of clot formation. For such investigations to take place, an *in vitro* setup that mimics the human circulation was developed. Different pulsatile shear rates were exposed to circulating blood which can readily induce variations in the shape, size, orientation, aggregate formation, and distribution of the erythrocytes. Furthermore, thromboplastin activation was achieved using a chemical trigger.

## Results

In this section, the measured values for fluid properties are presented, including the optical spectra in the visible and near-IR range, and viscosity measurements at varying shear rates. We further, present visual data from the collected signals; pressure, R and IR PPG signals at both stroke volumes and varying pumping frequencies in the arterial model. Furthermore, we present statistical analyses from the processed signals to draw average and standard variations of IR PPG_AC_ amplitudes, IR PPG_DC_ levels, mean pressure values, peak forward and reflected flow velocities along with multiple comparison tests. Finally, correlational studies were performed showing Rsquare values for the regression fitting models relating viscosity values with PPG_AC_ and PPG_DC_ for both wavelengths at varying pumping frequencies and before and after clot formations.

### Fluid properties

**Figure 1:**
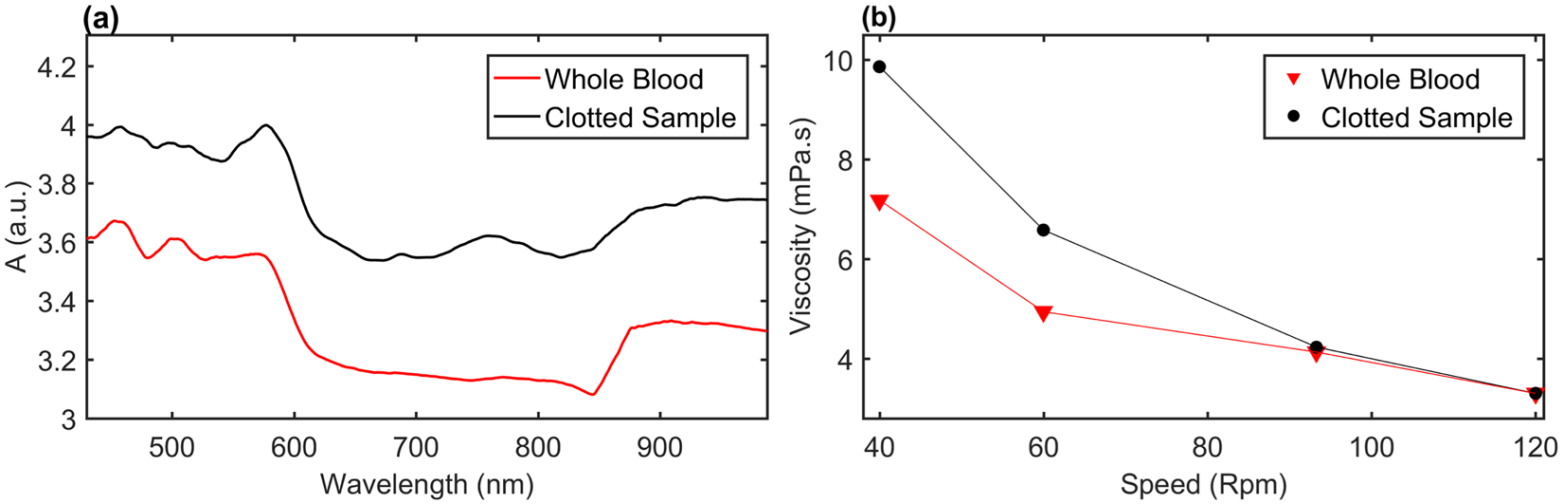
Optical Spectra and viscosity measurements. (**a**) Optical Spectra for a sample of whole equine blood and a fully clotted equine sample. (**b**) Shear controlled viscosity measurement, for whole equine blood and a fully clotted sample.

The haematocrit from five samples of whole equine blood was estimated using a graded 1 ml cuvette. The mark indicated a haematocrit average of 0.45 in all samples. The optical spectra in the visible and near-IR region for whole equine blood samples and clotted blood samples were obtained using a spectrometer (Fig. 1(a)). It is evident that clotted blood samples have higher absorption characteristics when compared to whole blood samples. Viscosity values at varying shear rates were measured using the cone/plate rheometer. Blood is known to inhibit shear thinning features, as seen in Fig. 1(b), where the viscosity decreases for both fluids with increasing shear rates. Moreover, clotted blood is shown to inhibit higher viscosity values than whole blood samples.

**Figure 2:**
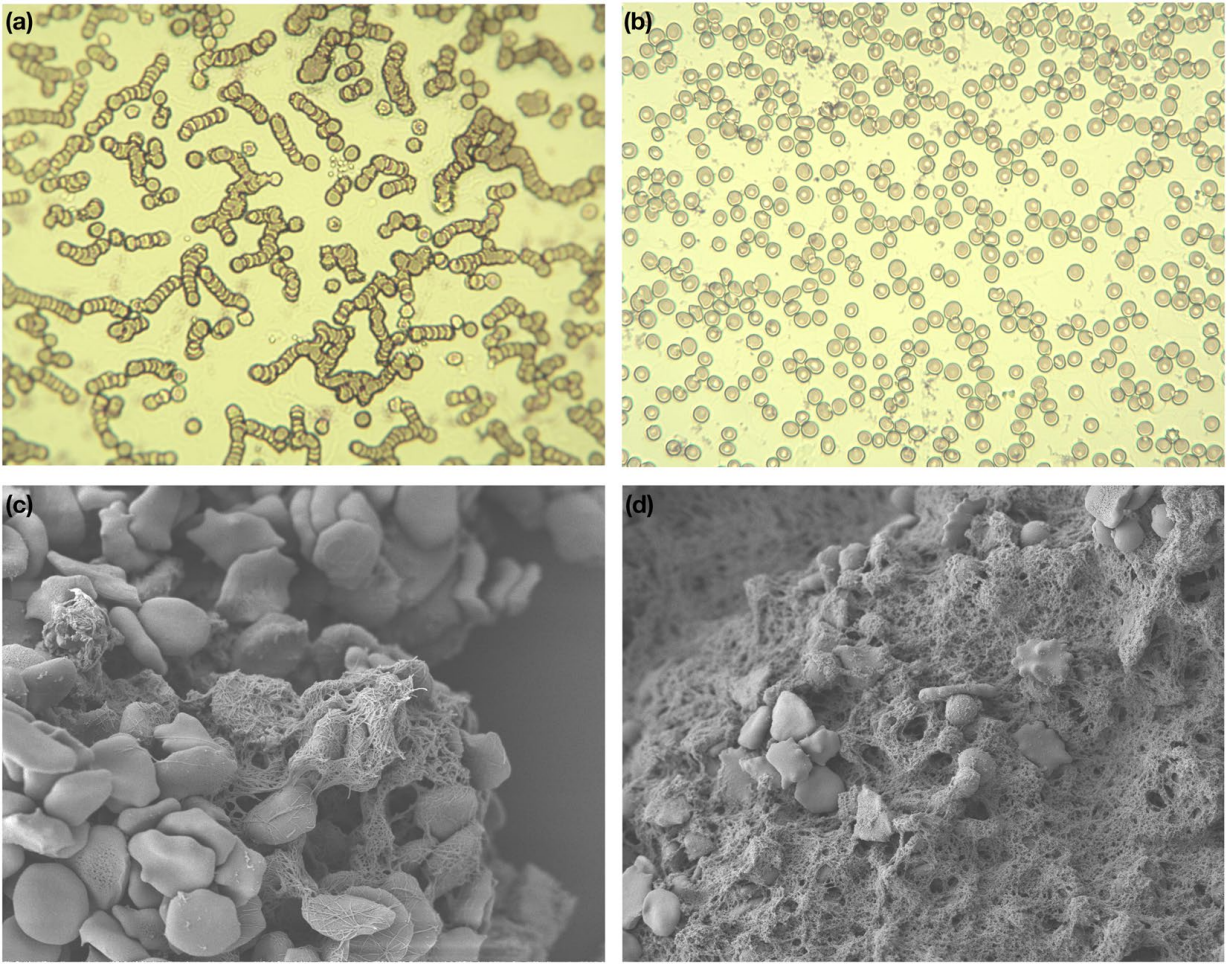
Equine blood microscopic images (**a**) showing aggregation of cells (coin-shape rouleaux formation) after samples were left to settle in the reservoir (magnification x400); (**b**) Sample images after pumping at varied shear rates (magnification x400); (**c**) Scanning Electronic Microscopic (SEM) images of a whole equine blood sample after two mins and; (**d**) SEM of whole equine blood sample after 20 mins of thromboplastin activation (magnification x5000), clearly showing erythrocytes *and the development of Fibrinous Matrix*.

Blood microscopic images of a stained equine sample are presented in Fig. 2, showing rouleaux formation from a sample left to settle at room temperature (see Fig. 2(a)) and the disaggregation of the erythrocytes after the sample was exposed to shear rates (see Fig. 2(b)), both obtained at a magnification of 400. Scanning Electronic Microscopy (SEM) images at a magnification of 5000 after two mins of thromboplastin activation are shown in Fig. 2(c) and after 20 mins of thromboplastin activation in Fig. 2(d). SEM images show the development of fibrinious matrices over the run of the clotting process.

### The role of red blood cells: Investigation of the effect of pumping frequency on the PPG signals

**Figure 3:**
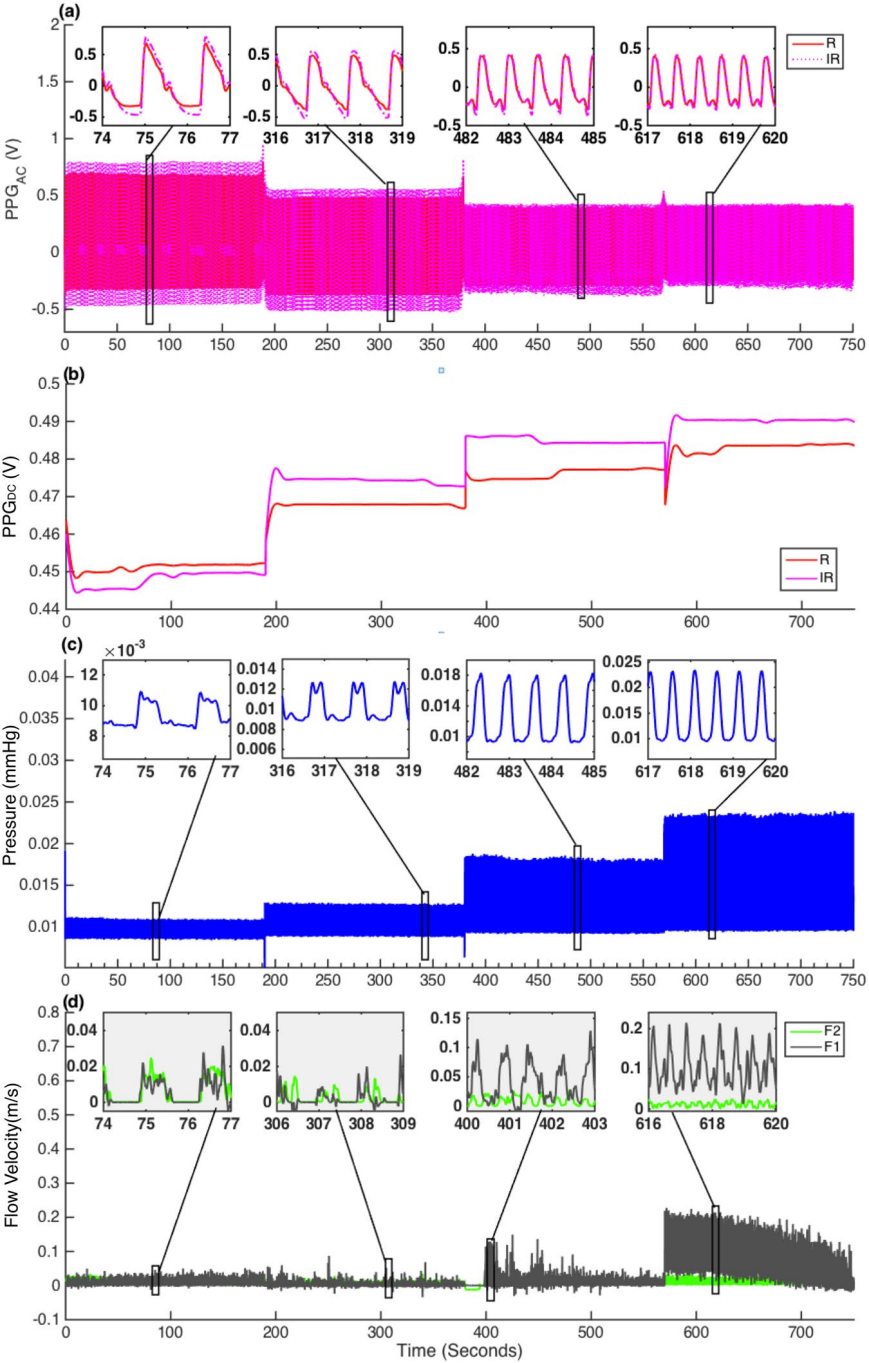
Signals collected during circulating whole equine blood. Data was obtained at 190 seconds for each pumping frequency (0.7, 1, 1.5 and 1.9 Hz). (**a**) Red (R) and Infrared (IR) PPGAC signals. (**b**) R and IR PPGDC levels. (**c**) Pressure (P) signals and (**d**) shows forward (F1) and backward (F2) flow velocities.

This section presents results obtained from circulating equine blood in the arterial model at varying pumping frequencies and stroke volumes. Visual representation of the collected data at varying pumping rates and a stroke volume of 70 ml are present in Fig. 3 for three minutes at each pumping frequency. Figure 3(a) shows the drop in R and IR PPG_AC_ amplitudes with increasing pumping frequencies, Fig. 3(b) shows the increase in R and IR PPG_DC_ levels with increasing pumping frequencies, Fig. 3(c) shows the increase in pressure values and Fig. 3(d) shows the behaviour of forward and backward flow velocities at the increasing pumping frequencies.

**Figure 4:**
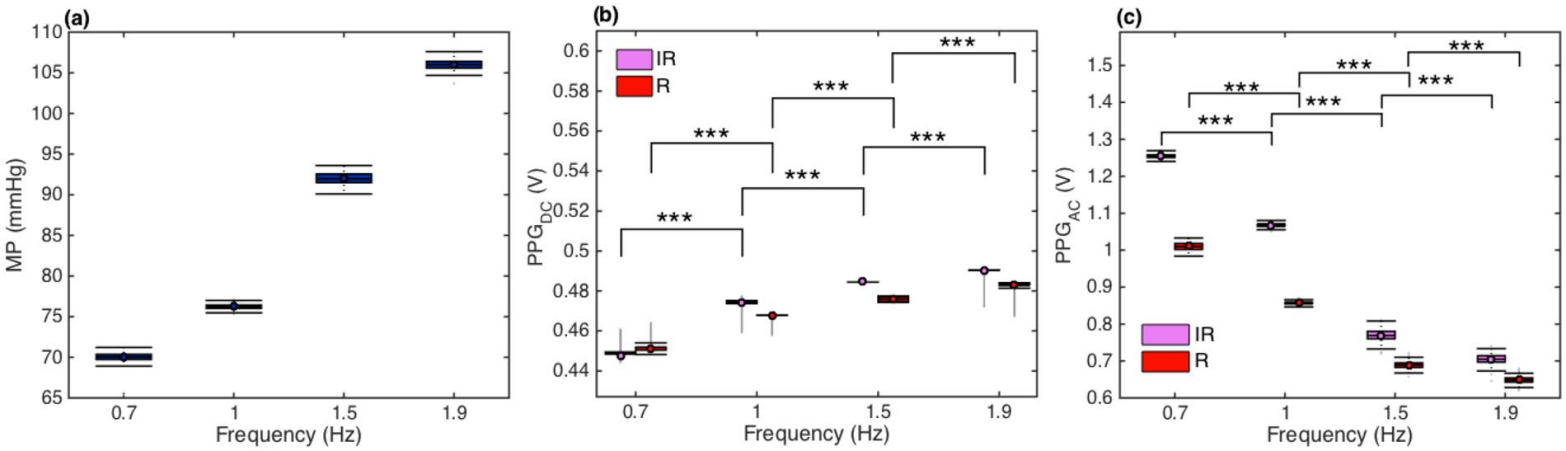
Boxplots for pressure and PPG components. Signals were obtained at varying pumping frequencies while circulating whole equine blood in a pulsatile laminar flow at a stroke volume of 70. (**a**) Mean pressure values, (**b**) R and IR PPGDC levels, (**c**) R and IR PPGAC amplitudes. P-values obtained from multiple Sidak’s test are displayed using the 3-star system. n = 760.

Statistics for mean pressure values averaged over 190 seconds at four different pumping frequencies while circulating equine blood are presented in Fig. 4(a). A highly significant increase in mean pressure values was observed with the increasing pumping frequencies. Analyses for DC levels for R and IR PPG signals averaged over 190 seconds at four different pumping frequencies are presented in Fig. 4(b). A highly significant increase was observed in PPG_DC_ levels for both wavelengths with increasing pumping frequencies. Red (R) and Infrared (IR) PPG_AC_ amplitudes averaged over 190 seconds at four different pumping frequencies are presented in Fig. 4(c). The highly significant drop was observed in AC amplitudes for both wavelengths with increasing pumping frequencies.

**Figure 5:**
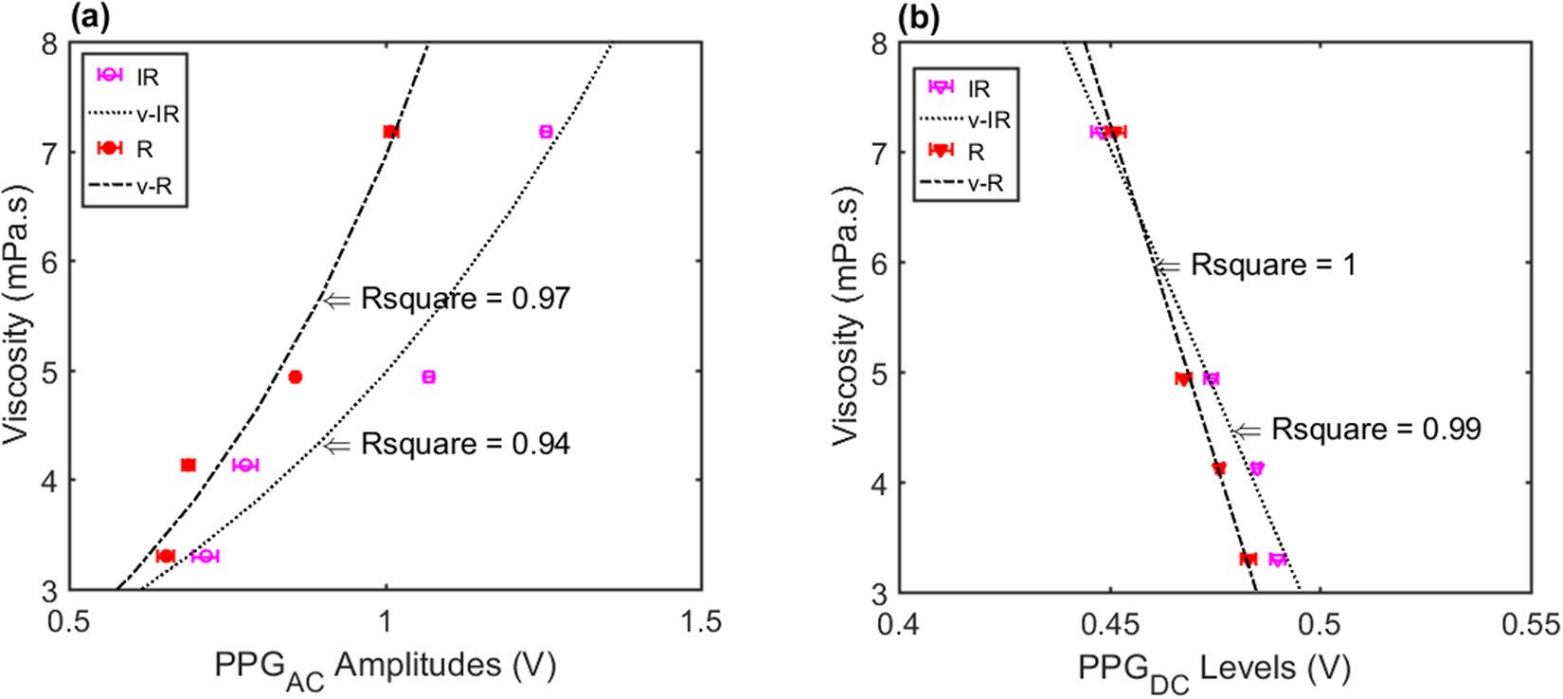
Regression models relating measured viscosity values with PPG components. Models created for R and IR PPG signals obtained from the *in vitro* experimental setup while changing pumping frequencies at 4 set-points. (**a**) Exponential regression model for viscosity values versus the amplitudes of PPG_AC_. (**b**) Linear regression model for viscosity values versus levels of PPG_DC_.

The relationship between viscosity values measured in the cone/plate viscometer and PPGAC and PPGDC are presented in Fig. 5. An exponential regression model provided the best fit for the viscosity-PPGAC amplitudes relationship with Rsquare of 0.97 for the R wavelength and 0.94 for the IR wavelength (Fig. 5(a)). A linear regression model was the best fit for the viscosity-PPG_DC_ relationship with Rsquare reaching 1 and 0.99 for R and IR respectively (Fig. 5(b)).

### The role of platelets: Investigation of the effect of thromboplastin activation on the PPG signals

**Figure 6:**
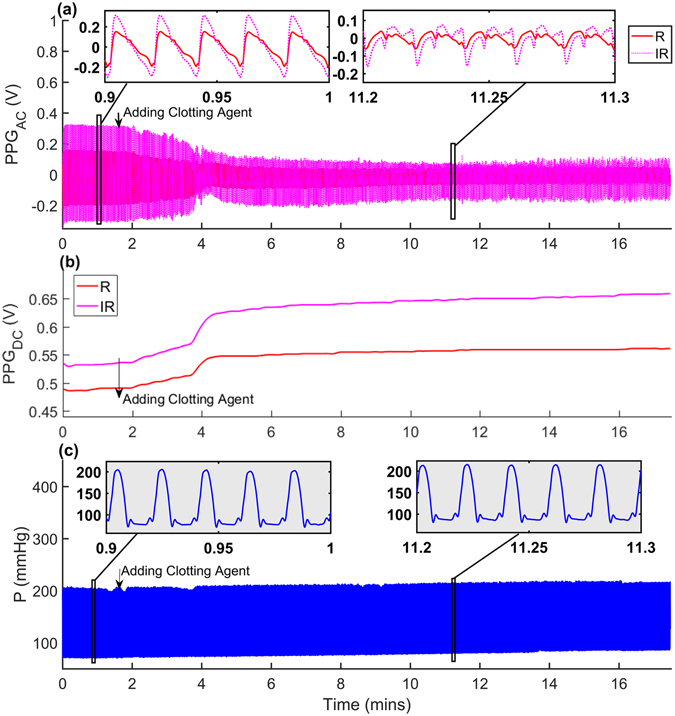
Collected signals before and after clot formations. Showing the signal behaviour after the clotting agent was introduced after 80 seconds of baseline data collection. (**a**) InfraRed (IR) PPG_AC_, (**b**) IR PPG_DC_, (**c**) pressure signals. The pumping frequency was set to 1 Hz and the stroke volume to 70 ml.

This section presents results obtained at baseline pumping rate of 1 Hz at a stroke volume of 70 ml in the arterial model. In this experiment, the recording continued for 20 mins after the clotting agent was added to the reservoir. The acquired signals are shown in Fig. 6. Both R and IR PPG_AC_ amplitudes, shown in Fig. 6(a), started to drop gradually once the chemical trigger was added, to finally stabilise after approximately 2.5 mins. The morphology of the signals was also significantly affected. In a similar way, both R and IR PPG_DC_ gradually increased once the chemical trigger was added to finally stabilise with higher values (Fig. 6(b)). Pressure values increased also gradually at the time of the addition of the chemical trigger, to finally stabilise at higher values (Fig. 6(c)).

**Figure 7:**
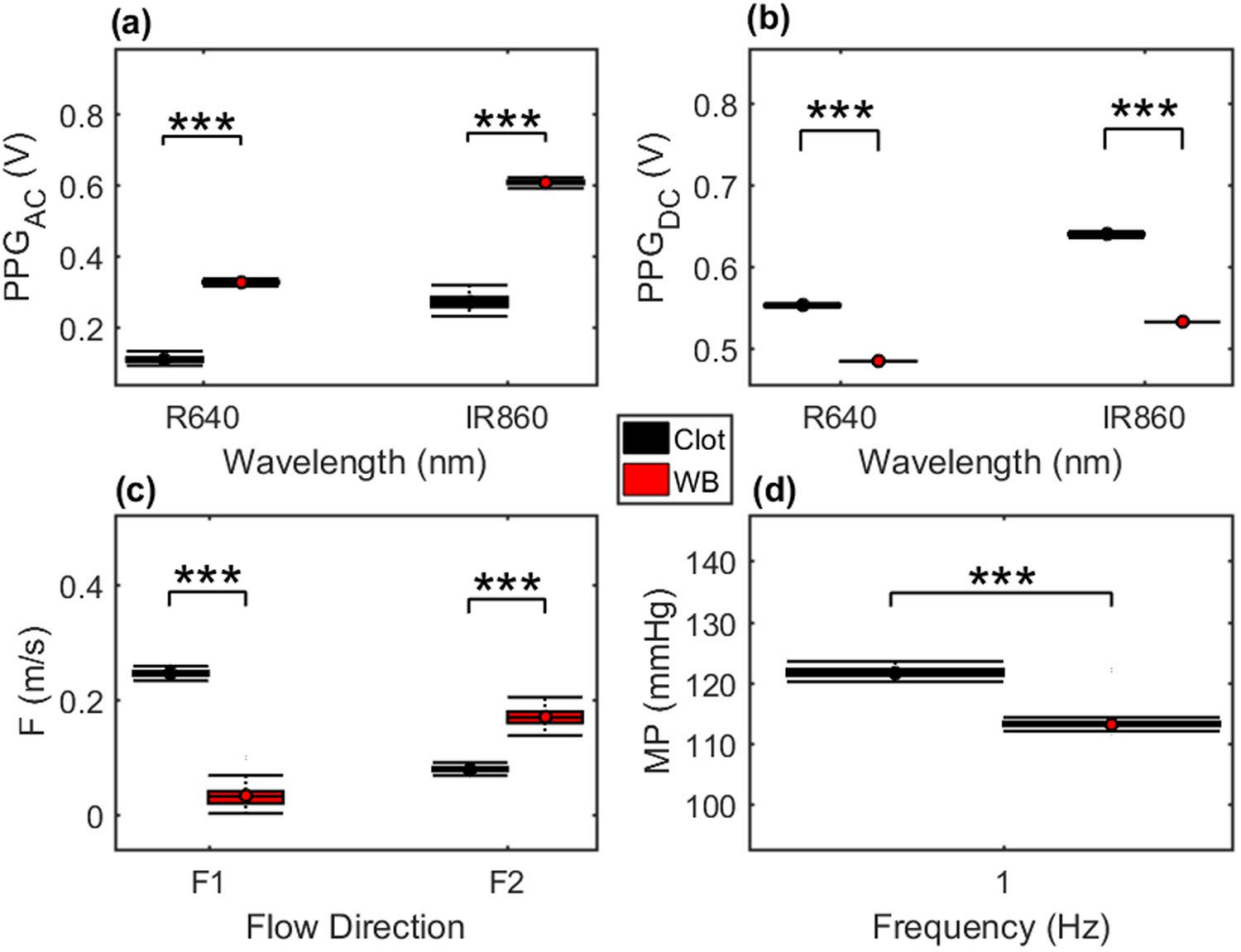
Statistical comparisons for PPG and haemodynamic data before and after clot formation. Data is obtained from the *in vitro* setup while circulating whole equine blood (WB) and after adding the clotting agent (Clot). Data obtained at a stroke volume of 70 ml and a frequency of 1 Hz. (**a**) R and IR PPG_AC_ amplitudes, (**b**) R and IR PPG_DC_ levels, (**c**) forward (F1) and backward (F2) flow velocities, and (**d**) mean pressure values. Statistical significance summary is displayed using the 3-star system.

Figure 7 shows statistical comparisons of values obtained from the *in vitro* setup while circulating whole equine blood (WB) and after adding the clotting agent (Clot). Analyses were performed over 80 seconds of baseline and 15 minutes of data obtained after total clotting at a stroke volume of 70 ml and a frequency of 1 Hz. Figure 7(a) demonstrates drop in R and IR PPG_AC_ amplitudes after clot formation, Fig. 7(b) shows the increase in R and IR PPG_DC_ levels after clot formation, Fig. 7(c) showing the increase in forward (F1) velocities and the drop in backward (F2) flow velocities after clot formation, and in Fig. 7(d) the increase in mean pressure values after clot formation is evident. Statistical significance is presented using the 3-star system.

**Figure 8:**
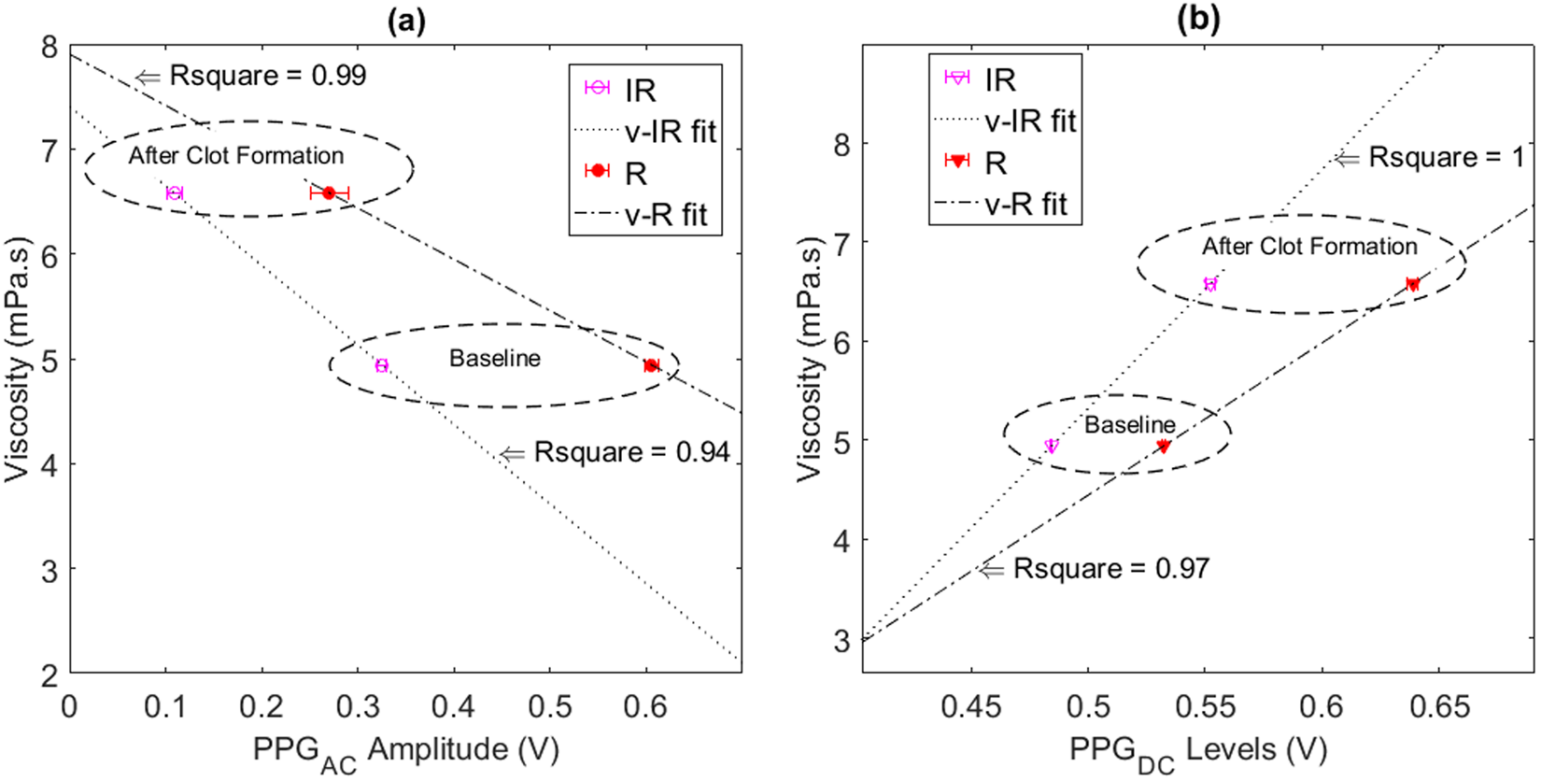
Regression models relating measured viscosity values with PPG components. Models created for R and IR PPG signals from the *in vitro* experimental setup before and after clot formation at a frequency of 1 Hz and a stroke volume of 70 ml. (**a**) Linear regression model for viscosity values (v) versus the amplitudes of PPG_AC_ at both wavelengths. (**b**) Linear regression model for viscosity values (v) versus levels of PPG_DC_ at both wavelengths.

In the same manner, as in stage 1, regression models relating blood viscosity values measured in the cone/plate viscometer with the PPG components before and after the clot formation while whole equine blood is being circulated in the *in vitro* setup at a frequency of 1 Hz and a stroke volume of 70 ml are displayed in Fig. 8. The linear regression model for the viscosity-PPG_AC_ relationship provided Rsquare of 0.99 for the R wavelength and 0.94 for the IR wavelength as seen in Fig. 8(a). While a linear regression model for the viscosity-PPG_DC_ relationship provided Rsquare of 0.97 and 1 for R and IR respectively are seen in Fig. 8(b).

## Discussion

The assessment of hemorheological properties is vital for the early diagnosis of pathologies, particularly cardiovascular diseases. Non-invasive continuous and real-time monitoring of haemorheology will contribute significantly in the detection/screening and perhaps monitoring of such diseases and it could find use in both clinical and home setting. This *in vitro* study investigates the potential of Photoplethysmography and its sensitivity in the detection of changes in blood optical characteristics under varied shear rates and during clot formations.

The first set of investigations explored the effect of pumping rates (0.67, 1, 1.5, 1.9 Hz) on the PPG components. It was observed that mean pressure values increased significantly (p < 0.001) with increasing pumping rates. This is an expected behaviour due to the increase in pumping power which is directly dependent on pumping frequencies. A highly significant (p < 0.001) drop in amplitudes of R and IR PPG_AC_ signals was also evident with increasing pulsating frequencies (See Figs 3(a) and 4(c)). At increasing shear rates, blood disaggregates and experiences a drop in viscosity values as seen in Figs 1(b) and 2(a,b). Both factors are expected to contribute to decreasing absorption properties. As a matter of fact, this is not completely an unexpected behaviour, and it is in good agreement with a previous investigation of the effect of shear rates on the optical properties of circulating blood^30^. Roggan and colleagues in their experiment showed that mean values of the absorption and reduced scattering coefficients at a haematocrit of 41% dropped significantly with increasing shear rates in a non-pulsating flowing scenario in a cuvette^30^. Roggan suggested that it is not clear if this behaviour is due to cell deformation or axial migration. Observing PPG_DC_ levels in Figs 3(b) and 4(b) provides a further indication to clarify these observations. Accompanied by the highly significant decrease in PPG_AC_ amplitudes, there was a highly significant (p < 0.001) increase in PPG_DC_ levels at elevated shear rates. DC levels can be seen as the light intensities resultant of the steady layer of the pulsatile flow that develops at the centre of the tube/artery and travels at the maximum velocity^31, 32^. The drop in PPG_AC_ and the increase in PPG_DC_ confirms that a major contributor to this behaviour is the radial migration of the erythrocytes towards the central layers of the flow.

The increase in PPG_DC_ levels might not be solely dependent on radial migration, which induces an increase in scattering due to the increase of cell count in that region. Moreover, it could be also related to the increasing shear rates and decline in fluid viscosities (Fig. 1(b)). Such changes will be accompanied with erythrocyte disaggregation and an increase in total flow velocities (Fig. 3). A process that will result in higher energy photon-flow collisions and a further increase in scattered light in the steady region. Hence, the increase in DC levels due to the almost perpendicular angle at the interface between the light beam and the steady layer. On the other hand, the expected disaggregation, increase in shear stresses and the drop in blood viscosity might also contribute to increased cell deformations as was recently documented^33, 34^. We speculate that this degree of deformation will cause the decrease in the scattering properties of the erythrocytes in conjunction with an increase in plasma gaps, as previously observed by Roggan and colleagues, and is especially evident in the oscillatory region as estimated with PPG_AC_ amplitudes. It is evident that PPG_AC_ components have strong correlations with changes in viscosity values, as seen in Fig. 5(a), where a nonlinear regression model provided Rsquare values of 0.94 and 0.97 for R and IR, respectively. While a linear regression model provided Rsquare values of 0.99 and 1 for R and IR PPG_DC_ (Fig. 5(b)), respectively.

Finally, we explored the effect of platelet activation on both optical and fluid dynamic signals. The chemically triggered platelet activation was achieved using calcium chloride. Clot formation is initiated by Ca^2+^-dependent binding of the coagulation factor VIIa (FVIIa) to its cofactor, tissue factor (TF). FVIIa complex activates factors IX and X, ultimately leading to the formation of thrombin and the coagulation of blood. Thrombin catalyses the conversion of fibrinogen molecules to fibrin. The polymerisation of fibrin in vessels gives rise to thrombus formation^35^. Microscopic images, seen in Fig. 2(c), confirmed the initiation of the formation of the fibrinous matrix (cross-linking fibrin) at two mins and the continuing of the fibrinous formation where the sample was completely covered with cross-linking fibres after 20 minutes as seen in Fig. 2(d). While circulating the clotting blood, mean pressure values increased significantly after the clotting activation (see Fig. 7(d)), which can be referred to the increase in viscosity of the clotting sample (see Fig. 1(b)). The PPG_AC_ amplitude started to decrease (see Fig. 6(a)), a few seconds after the clotting agent was added. This drop continued gradually until total clotting of the whole sample was achieved. At that instance, PPG_AC_ amplitudes stabilised with significantly (p < 0.001) lower amplitudes and with disrupted morphology. This behaviour can be explained by observing the acquired optical spectra in the R and IR regions seen in Fig. 1(a). The clotted blood sample had higher absorption than that observed in whole blood, a behaviour that can be referred to the formation of fibrinous matrices seen in the microscopic images in Fig. 2(d). We also observed the change in the morphology of PPG_AC_ signals indicating a significant change in the scattering properties of the medium. The development of fibrinous matrix increases the absorptivity of whole blood and shield the optical scattering of individual cells causing the disruption to the morphology of the PPG_AC_ signal. R and IR PPG_DC_ levels seen in Fig. 7(c), increased significantly (p < 0.001) after the introduction of the clotting agent. This response might be related to the increase in forward flow velocities and pressure values and could be associated with changes in the elastic properties of the erythrocytes. Regression models seen in Fig. 8 show strong correlations between both PPG components with changes in viscosity before and after the addition of the clotting chemical trigger. Nevertheless, it is important to highlight that the underlying cause of changes in viscosity need to be considered, and the way shear rates affect the PPG signal are different from that observed during clot formations. This is due to the absence of fibrinous matrices which can be reflected in the optical properties of whole blood samples.

Despite that this study has demonstrated a clear proof of concept and paves the way for how Photoplethysmography can reflect changes in the rheological properties of blood in relation to changes in shear rates or clot formations, it will be an omission not to identify some of the limitations. The main limitation is the nature of the study, meaning *in vitro*. The developed *in vitro* model was only a tool to provide a proof of concept, hence, only the arterial component of the system was presented and the compliance, single diameter and blood oxygenation are limiting factors. PPG signals obtained *in vivo* will contain physiological information related to respiration, temperature changes, nitric oxide release, vasoconstriction and dilation, amongst others. Hence, an effective and systematic PPG decomposition technique together with rigorous *in vivo* studies will be required in order to isolate and account the contributions of such parameters on the PPG signal prior to validation of the PPG in the detection of blood viscosity and clot formation non-invasively in human subjects. Moreover, age, arterial calcification and stiffness, hypertension are also factors that can affect the PPG signal, and it will be crucial to investigate the PPG signal in relation to haemodynamic changes in order to separate the effect of such factors. Last but not least, it is generally known that the PPG signal is not yet standardised due to its sensitivity to skin absorption properties, blood oxygenation levels, geometrical and mechanical properties of vessels and blood. Nonetheless, this need not be a disadvantage but a challenge to those that are engaged in PPG research, where through rigorous *in vitro*, *ex vivo* and *in vivo* investigations aiming to fully understand and “map” the contributions of the PPG signal to various haemodynamic, vascular mechanics and hemorheological changes will lead to major contributions in healthcare.

## Materials and Methods

### PPG sensors and processing system

**Figure 9:**
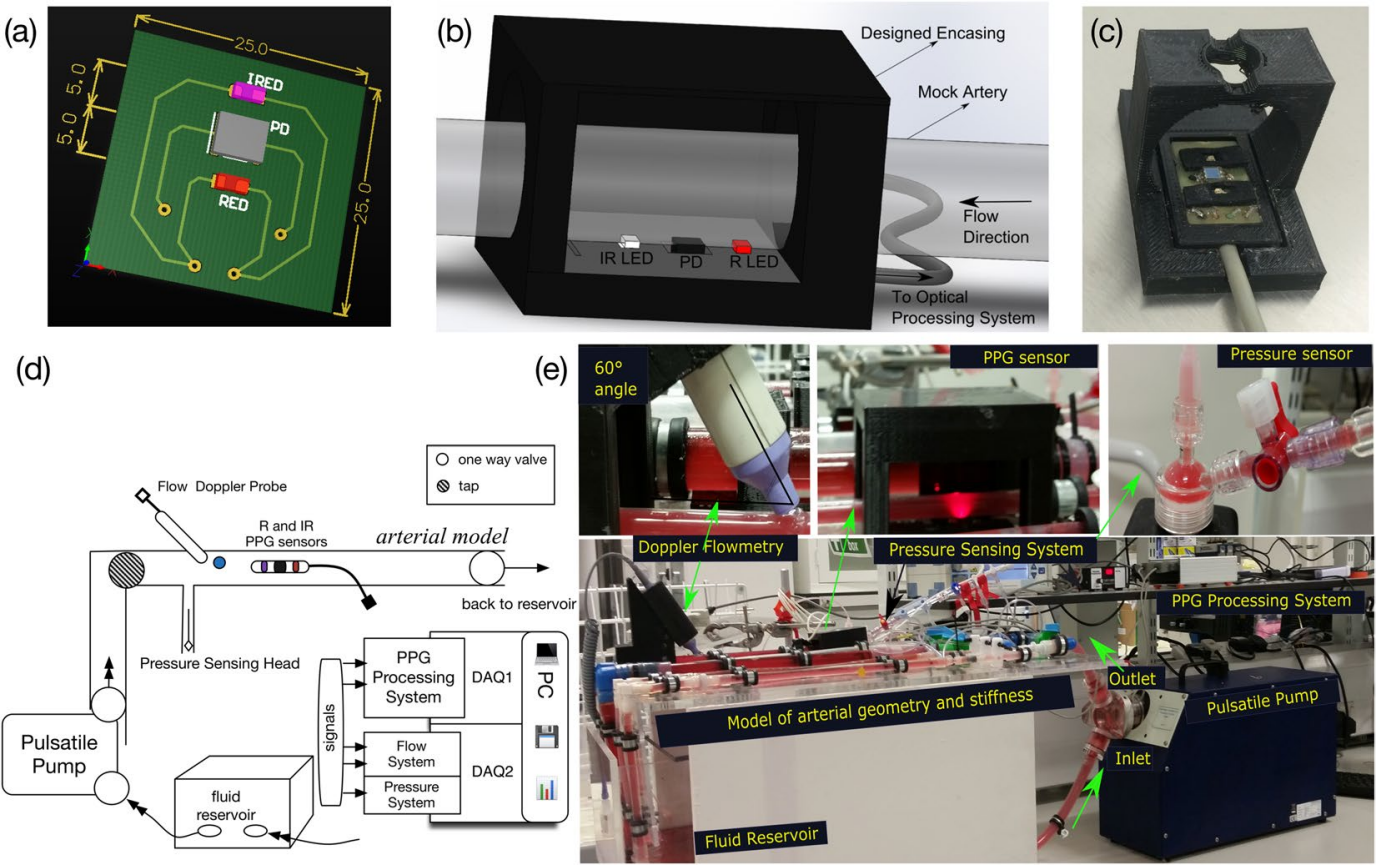
*In vitro* setup design and implementation. (**a**) Sensor PCB design, (**b**) 3D model of sensor and encasing, (**c**) close-up picture of inner encasing and sensor, (**d**) schematic of the experimental setup (**e**) picture of the final experimental design.

Reflectance PPG sensors were designed and fabricated as seen in Fig. 9(a–c). Two LEDs, Red (R) and Infrared (IR), at a peak wavelength of 640 nm and 860 nm respectively, were aligned in a reflectance mode at a five mm distance from the photodiode (PD) (Vishay, UK). A piece of black rubber was inserted between the LEDs and the photodetector to avoid saturation of the photodiode. A 3D encasing was designed and printed (Makerbot, US) to fix the probe at one mm contact gap with the pulsating tube without interfering with its motion. For the acquisition of PPG signals, a custom-made two-channel dual wavelength PPG instrumentation system was designed. The PPG processing system was constructed to pre-process and convert the detected current into voltages for later acquisition. The processing system used multiplexed current sources (set to 25 mA) to drive both R and IR LEDs consecutively. A microcontroller generated the digital switching clock so that the photodetector captured both components at a frequency of 900 Hz. The mixed signals were fed into the demultiplexer and were then split into their respective R and IR PPG signals. PPG signals were digitised using the PCIe-6321 (DAQ1) (National Instruments, UK) at a sampling rate of one kHz.

### Blood

Equine blood (TCS Biosciences Ltd, UK) was used. The blood was pooled and treated with the anticoagulant Acid Citrate Dextrose (ACD) immediately after being collected from donors as advised by the supplier. Equine blood was chosen due to its properties that are similar to human blood. Equine red blood cells are biconcave discs of dimensions 6–7 $\mu$m diameter, 2.5 $\mu$m thick at the edge and 1 $\mu$m thick at the centre. An important feature is the ability of equine blood to form rouleaux, a property that is present in human blood and not in other species (i.e. Bovine). The optical spectra were obtained using a sample of whole equine blood in R and near-IR regions using the spectrometer (Lambda1050, PerkinElmer, US). Viscosity measurements were obtained using the cone/plate rheometer (Brookfield, DV-III ultra, US). Haematocrit values were estimated using a centrifuge (Sorvall™ RX1 Thermo Scientific) to separate red blood cells from other components in a graduated microtube. Slide microscopy was prepared using the Wright-Giemsa staining method and microscopic images of whole equine blood samples were obtained at a magnification of 400 (Microtec RM-1, TEC Microscopes LTD). During the clot-formation investigation, thromboplastin activation was tested using calcium chloride (24 g/L CaCl_2_) on a 10 ml whole equine sample^36^. Scanning Electronic Microscopic images were obtained to confirm the activation. The images were captured after sample fixations using the following protocol: three drops of clotted equine blood samples were added to 5 ml of 2.5% glutaraldehyde in 0.1 M cacodylate buffer at pH 7.4. Samples were fixed overnight at room temperature. Blood cells were washed twice with cacodylate buffer. The samples were dehydrated in ascending concentrations of ethanol up to absolute. Samples were transferred to pure dry acetone and suspended in a final change of acetone-washed coverslip and then gold-coated in a Polaron sputter coater. The samples were photographed on fine grain film at ×5000 in the Autoscan electron microscope at 20 KV.

### *In vitro* setup and protocol

A pulsatile flow loop illustrated in Fig. 9(d) was designed to contain an elastic tube model that produces a range of patterns of mechanical forces at the inner surface of the flexible tubing. A pulsatile pump (1423 PBP, Harvard Apparatus, US) was used to generate controlled pulsatile flow. A custom-made Plexiglas fluid reservoir was fabricated to contain the fluid within the circulation. A flexible PolyVinyl Chloride (PVC) tubing (length: 20 cm, Outside Diameter (OD): 22 mm, and an Inner Diameter (ID): 16 mm) was connected to the input of the system to enable a fully developed flow before entering the model. The model consisted of a PVC tube (Elastic Modulus ~23 MPa, ID: 16 mm, OD: 1.8 mm) which simulates a large artery. The model was mounted onto a specially designed support system which incorporated rubber clamps to hold the tubes at a constant length without interfering with their movement. An initial axial stretch of 1–2% was used to ensure that the flexible tubes remained in a straight position during the pumping phase. One-way check valves with pre-set opening pressure (50 mmHg) were introduced at both ends of the model which provided the control over the resistance and reflected flow. Valves were also added at the entrance of the design to allow control of flow paths and switching to the bypassing tube in order to eliminate any bubbles in the system. A picture of the final experimental design is seen in Fig. 9(e).

Two litres of equine blood were circulated in the arterial model at ambient temperature (~23 °C). The reservoir consisted of a magnetic stirrer spun at low speed to prevent sedimentation. The blood was pumped from the blood reservoir to the arterial model maintaining laminar patterns with no evidence of haemolysis. The experiment was performed in the following steps: The blood was circulated at a stroke volume of 70 ml for two minutes to allow the system to stabilise. Data collection started at a pumping frequency of 40 bpm, and the recording continued for 10 minutes. Pumping frequency was increased to 60 bpm, 80 bpm and then 90 bpm for 10 minutes at each speed. The recording stopped, and the blood was left to regain rheological properties for an average of 10 minutes while the magnetic stirrer was operating to prevent any sedimentation. Microscopic images were obtained to confirm recovery of rheological status. The pumping speed was set back to 1 Hz, and the system resistance was increased, and the recording started again for 80 seconds. The clotting agent (CaCl_2_ at a concentration of 24 g/L) was added and the pump was left to operate for 20 minutes while data collection continued. Finally, the pump was emptied, washed, sterilised and left to dry.

### Blood pressure and flow velocity measurements

Blood pressure and flow velocity measurements were obtained to map the haemodynamic forces throughout the experiments. Pressure measurements were acquired using a research grade pressure transducer (Harvard Apparatus, US). Flow velocity was measured at the centre of the arterial model using the MD2 ultrasound Doppler (Huntleigh Healthcare, UK) with an 8 MHz probe. A 3D printed holder was designed to fix the probe at a 60° angle with the tube without interfering with the wall motion as seen in Fig. 9(e). Pressure and flow velocity signals were digitised using the 9172-c Data-Acquisition card (National Instruments, US) at 1 kHz sampling frequency. While the focus of this study is the effect of haemorheology on the PPG components, pressure and flow velocity measurements were obtained to aid the general understanding of the flow dynamics during the experiment. It is also worth mentioning that flow velocity signals might incur limitations due to the contact of the probe with the tube due to the change in pulsating frequency.

### Data collection and statistical analyses

The collected data via the LabVIEW Virtual Software (National Instruments, US) were analysed in an offline custom written script using MATLAB (Mathworks, US). The obtained data were pressure signals, forward and reflected flow velocities, IR and R, PPG_DC_ and PPG_AC_ signals. The data was grouped to include values at varying pulsating frequencies at the first stage and to compare values before and after introducing the clotting agent in the second stage. Statistical analyses were performed to compare values at different pulsating frequencies for each model. The data was confirmed to follow a Gaussian distribution. When multiple comparisons were performed, a one-way ANOVA test was used. This was followed by Sidak’s multiple comparison tests to evaluate significance. A two-tailed t-test were also used to compare two groups only (before and after clot formations). The significance is displayed on graphs using the star system where ***for p < 0.001, **for p < 0.01 and *for p < 0.05. The correlation studies between the PPG components and the measured viscosity values were also performed using linear and non-linear regression models and the goodness of fit is stated with the Rsquare value.
